# The ground beetle genus *Bembidion* Latreille in Baltic amber: Review of preserved specimens and first 3D reconstruction of endophallic structures using X-ray microscopy (Coleoptera, Carabidae, Bembidiini)

**DOI:** 10.3897/zookeys.662.12124

**Published:** 2017-03-21

**Authors:** Joachim Schmidt, Peter Michalik

**Affiliations:** 1 University of Rostock, Institute of Biosciences, General and Systematic Zoology, Universitätsplatz 2, 18055 Rostock, Germany; 2 University of Marburg, Fb. 17 - Biologie, Karl-von-Frisch-Straße 8, 35043 Marburg, Germany; 3 Zoological Museum, University of Greifswald, Loitzer Str. 26, 17489 Greifswald, Germany

**Keywords:** Ground beetles, *Archaeophilochthus*, *Eodontium* new subgenus, new species, 3D reconstruction, Eocene, Christian Gottfried Giebel collection

## Abstract

The ground beetle genus *Bembidion* is a highly diverse group of small predators with more than 1.200 described extant species. In contrast, only two representatives of *Bembidion* are known from the amber fossil record and their position within this mega-diverse genus is dubious. Here, we address the taxonomic position of these two extinct *Bembidion* species (*B. succini* Giebel, 1856 and *B. christelae* Ortuño & Arillo, 2010). Based on the insufficient description and the missing type specimen, *B. succini*, nomen dubium, cannot be assigned to the genus *Bembidion* and/or to the tribe Bembidiini with certainty. The subgenus Archaeophilochthus Ortuño & Arillo, 2010 was erected for the second extinct species, *B. christelae*, based on external characters. However, this species seems indistinguishable to members of the earlier described subgenus Philochthemphanes Netolitzky, 1943 which comprises about extant 10 species distributed in East and Southeast Asia. Furthermore, we describe two new species, *B. bukejsi* **sp. n.** and *B. alekseevi* **sp. n.**, from the Eocene Baltic amber using X-ray microscopy. Based on external and genital morphology including endophallic structures, we erected the monotypic subgenus Eodontium **subgen. n.** for *B. bukejsi* **sp. n.**, which is probably related to the subgenera *Andrewesa* Netolitzky, 1931, the *Hydrium* complex, or the *Odontium* series sensu Maddison (2012). On the other hand, *B. alekseevi* **sp. n.** can be assigned to the subgenus Eupetedromus Netolitzky, 1911. The occurrence of representatives of at least two species groups adapted to a temperate climate suggests the presence of at least locally temperate climates in Baltic amber forests.

## Introduction

Ground beetle fossils of the mega-diverse genus *Bembidion* preserved in Baltic amber are frequently mentioned in catalogues of amber inclusions (Klebs 1910, Bachofen-Echt 1949, Larsson 1978, Spahr 1981, Keilbach 1982, Hieke and Pietrzeniuk 1984, Alekseev 2013). However, until today only two *Bembidion* inclusions are described to species level: *B. succini* Giebel, 1856, and *B. christelae* Ortuño and Arillo, 2010. A careful examination of the amber fossils preserved at several institutional collections in Germany performed by one of us (J.S.) revealed that previous identification are often erroneous and confused with species of other ground beetles lineages, mainly Tachyina and Trechini, whereas true *Bembidion* fossils seem very rare inclusions. Fortunately, we found two specimens of *Bembidion* well preserved in Baltic amber and housed in the Andris Bukejs Collection (Daugavpils) and in the Vitalii I. Alekseev Collection (Kaliningrad) available for a detailed study using light and X-ray microscopy. Both specimens are representatives of hitherto unknown species. In this paper we describe these new taxa in detail and address their taxonomic relationships. Furthermore, we address the taxonomic position and history of the two previously described *Bembidion* fossils. The current knowledge regarding the location of the amber fossil collection of Christian Gottfried Giebel is summarized. Finally, based on the habitat preferences of extant *Bembidion* lineages related to the fossil lineages, we hypothesize some ecological conditions likely present in the Baltic amber forests of the Eocene North Europe.

## Material and methods

The specimen was studied and imaged using light microscopy and micro-CT. The methods and technology used were described in detail in previous works by Schmidt et al. (2016a, b). Additionally, we reconstructed the endophallic structures of *B. bukejsi* sp. n. by delineating the structures of interest in each virtual section using the segmentation editor in Amira 5.4.5. The image stacks of the micro-CT scans have been deposited in MorphDBase (https://www.morphdbase.de).

Measurements of the fossil specimen were taken as follows: body size was quantified by the standardized body length, i.e., the sum of: (1) the distance from the apex of the right mandible in closed position to the cervical collar, (2) the median length of the pronotum, (3) the distance from the base of the scutellum along the suture to the apex of the left elytron. The width of the head, of the pronotum, and of the elytra was measured at their widest points. The width of the pronotal apex was measured between the tips of the apical angles, the width of the pronotal base was measured between the tips of the laterobasal angles.

## Taxonomy

### Bembidion succini

Taxon classificationAnimaliaColeopteraCarabidae

Giebel, 1856 , nomen dubium

Bembidium succini Giebel, 1856: 64.

#### Remarks on description and type material.

*B. succini* was described from Baltic amber and it is the first fossil species described in this genus to species level. Although it was mentioned in catalogues of Baltic amber fossils (Spahr 1981, Keilbach 1982, Alekseev 2013) subsequent researchers did not discuss the taxonomic position of this species.

The original description of *B. succini*, however, does not provide information to which genus of ground beetles this species actually belongs. Giebel (1856) noted that he was unable to recognize the diagnostic characters of the genus as well as of the whole Bembidiini tribe in this species. Mouth parts, the ventral side of body, elytral apex, and the legs were not visible to him, or he did not describe relevant characters of these body parts. The placement of this fossil in the genus *Bembidion* was solely based on the similarity of the external shape and proportions compared to some extant Central European species of the subgenus Ocydromus Clairville, 1806. The following citation represent the complete description provided by Giebel (1856):

„Das einzige Bernsteinexemplar der Leipziger Universitätssammlung ist kaum eine Linie lang und nähert sich zunächst den lebenden *B. brunnicorne*, *B. perplexum*, ist jedoch noch schmäler und gestreckter als diese, das Halsschild mit weniger convexen Seiten, die Flügeldecken mit feinen Punktstreifen, das ganze Tierchen hellgrün. Leider umgibt eine Blase das Thierchen so, daß ich weder die Beine noch die Palpen deutlich erkennen kann und nur aus den übrigen Formverhältnissen auf die Gattung *Bembidium* schließe“ (Giebel 1856: 64).

Based on the few character states presented in this description it cannot be excluded that the name *B. succini* is given to a tiny (body length not even 2.3 mm) representative of the subtribe Tachyina or even to an Eocene species of a non-Bembidiini lineage.

Unfortunately, the taxonomic position of *B. succini* has remained ambiguous since all Baltic amber fossils of the Christian Gottfried Giebel collection are missing today. About 150 years ago, the fossil collection of Giebel was completely moved from the palaeontological collections of the Leipzig University to the University of Halle where it is now part of the geoscientific collections of the Institute of Geosciences and Geography. In 1973 parts of this collection were loaned to the Bulgarian Academy of Sciences for further study and were probably returned to Halle in 1998, however, details of the transfer of the material as well as its current location are unknown (Henniger 2011). It is also unknown whether the loaned material contained amber fossils. In any event, nowadays not a single amber fossil ex collectio Giebel exists in the University of Halle (Norbert Hauschke, Institute of Geosciences and Geography, University of Halle, pers. comm.)! Thus, at the current state of knowledge it remains unclear whether the amber fossils of the Giebel collection are fully lost or only stored well hidden, e.g., within sealed containers in the stack-rooms of the University of Halle (Henniger 2011).

### Bembidion christelae

Taxon classificationAnimaliaColeopteraCarabidae

Ortuño & Arillo, 2010

Bembidion (Archaeophilochthus) christelae Ortuño & Arillo, 2010: 190.

#### Remarks on taxonomic position.

A monotypic subgenus Archaeophilochthus Ortuño & Arillo, 2010 was established for the fossil *B. christelae*. In the subgeneric diagnosis, the authors mentioned the following six features to be important for assigning the taxon: (i) elytral discal setae each are inserted in the third interval separated from the third stria; (ii) the groove of the rounded humeral margin ends “close to the 4^th^ or 5^th^ striae” (Ortuño and Arillo, 2010: 191, subgeneric diagnosis) and “at the base of 6th interstriae” (p. 190, species diagnosis), respectively; (iii) humeral setae of the elytral umbilicate series grouped and +/- equidistant; (iv) pronotal basolateral fovea poorly delimited; (v) pronotal laterobasal angles with carina well developed; vi) pronotal laterobasal angles formed oblique.

Because the first five of these features are similarly developed in *Philochthus* Stephens, 1828, Ortuño and Arillo (2010) assume a close relationship of the fossil with this Bembidion subgenus. Character state (vi) was considered plesiomorphic by the authors, and because the apomorphic state ‘pedunculated pronotal basal margin’ is developed in Philochthus the authors proposed a separate subgenus for the B. christelae.

Two extant *Bembidion* subgenera have been identified to be closely related to *Philochthus* (Maddison 2012) and which should be therefore considered when discussing the probable relationships of *B. christelae*: *Lindrochthus* Maddison, 2012, and *Philochthemphanes* Netolitzky, 1943. The former is monotypic and only includes *B. wickhami* Hayward, 1897, a species endemic to north-western North America. *Lindrochthus* is distinguished from *Philochthus* “by the less abruptly sinuate hind margin of the prothorax, and the reduced number of elytral striae” (Maddison 2012: 570). The pronotal margin is, however, more markedly sinuate in *B. wickhami* than in the fossil *B. christelae*, and the latter possesses eight completely developed elytral striae (Ortuño and Arillo 2010), which represents the plesiomorphic state. *Philochthemphanes* Netolitzky, 1943 (type species: *B. exquisitum* Andrewes, 1923 from the Central Himalaya) is a Bembidion subgenus containing little more than ten species distributed in East and Southeast Asia (Lorenz 2005, Toledano 2008, database of J.S.) and is considered to be the sister group of *Philochthus* based on molecular data (Maddison 2012). Species of *Philochthemphanes* possess a moderately sinuate pronotal basal margin as developed in *B. christelae*. Most noteworthy, *B. christelae* shows all diagnostic features of the exoskeleton compared to *Philochthemphanes*. It is thus very likely that the fossil taxon *Archaeophilochthus* is a junior synonym of *Philochthemphanes*. This assumption is further supported biogeographically as the flora and fauna of the Baltic amber forests is very similar to the modern biota of Indo-Malaya where *Philochthemphanes* is distributed nowadays (Wolfe 1975, Schmidt 2015). Here, extant species of *Philochthemphanes* inhabit cloud forests and forest’s edges and the adults can be found on the ground as well as on the mossy branches of shrubs and trees, even though these species lack the common features present among most of the arboreal ground beetles. Therefore and in contrast to the suggestion of Ortuño and Arillo (2010), *B. christelae* maybe had a semi-arboreal life style very similar to extant *Philochthemphanes* species. On the other hand, *Philochthemphanes* is not defined by clear synapomorphies. Thus, a careful revision and character analysis of *Philochthemphanes* is needed before a well-funded taxonomic conclusions regarding the synonymization of *Archaeophilochthus* and *Philochthemphanes* can be drawn.

### Bembidion bukejsi sp. n.

Taxon classificationAnimaliaColeopteraCarabidae

http://zoobank.org/661D694B-E65E-4D6F-A0D6-A26C0BC3D50E

Figs 1–2 , 3–5 , 6–8 , 9–13 , 14–17 , 26 , 27

#### Holotype.

Male in Baltic amber; size of amber piece approximately 8.9 × 4.7 × 2.5 mm (Fig. 2), with collection label data “012”, in Andris Bukejs collection, maintained at Institute of Life Sciences and Technologies, Daugavpils University (Daugavpils, Latvia). This amber piece was found elsewhere in the area of the Curonian Spit.

**Figures 1–2.**
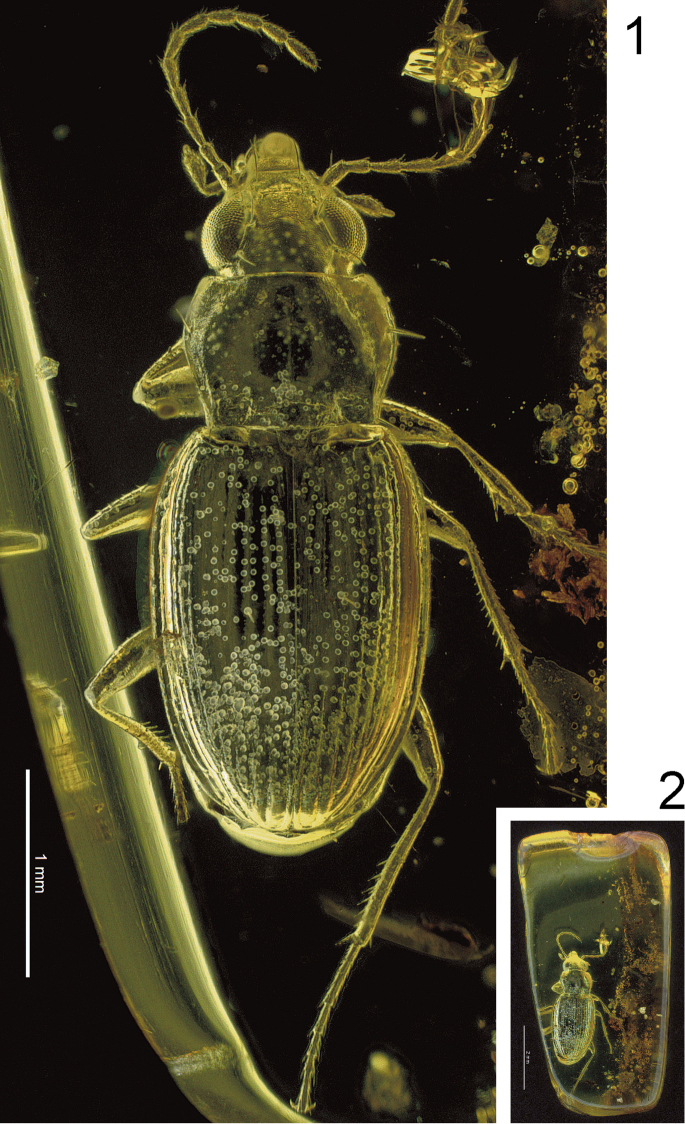
*Bembidion bukejsi* sp. n., light microscopic images of the holotype. **1** dorsal aspect **2** general view of the fossil with contours of the amber piece.

#### Preservation status.

**Figures 3–5.**
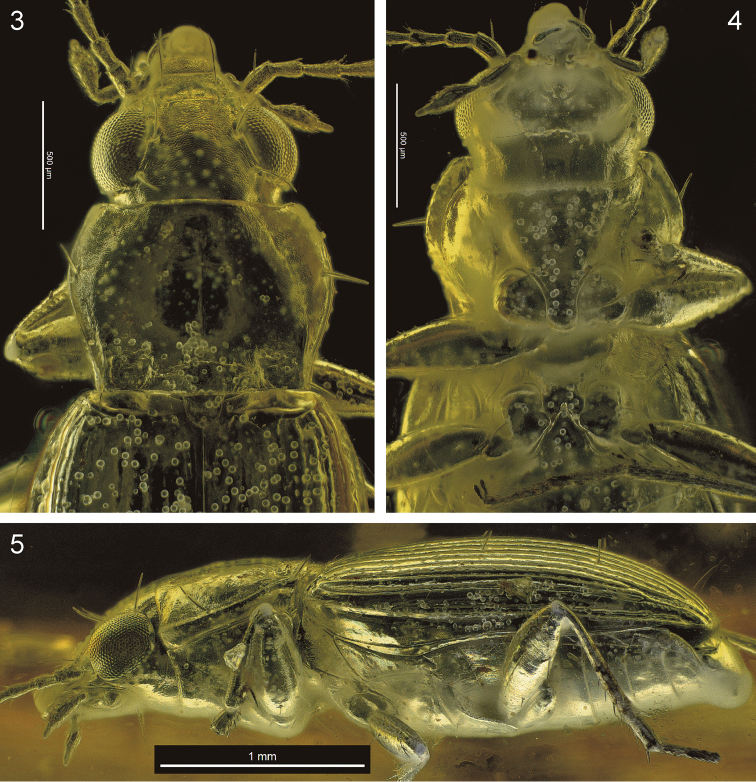
*Bembidion bukejsi* sp. n., light microscopic images of the holotype. **3** anterior portion of body, dorsal aspect **4** anterior portion of body, ventral aspect **5** left lateral aspect.

**Figures 6–8.**
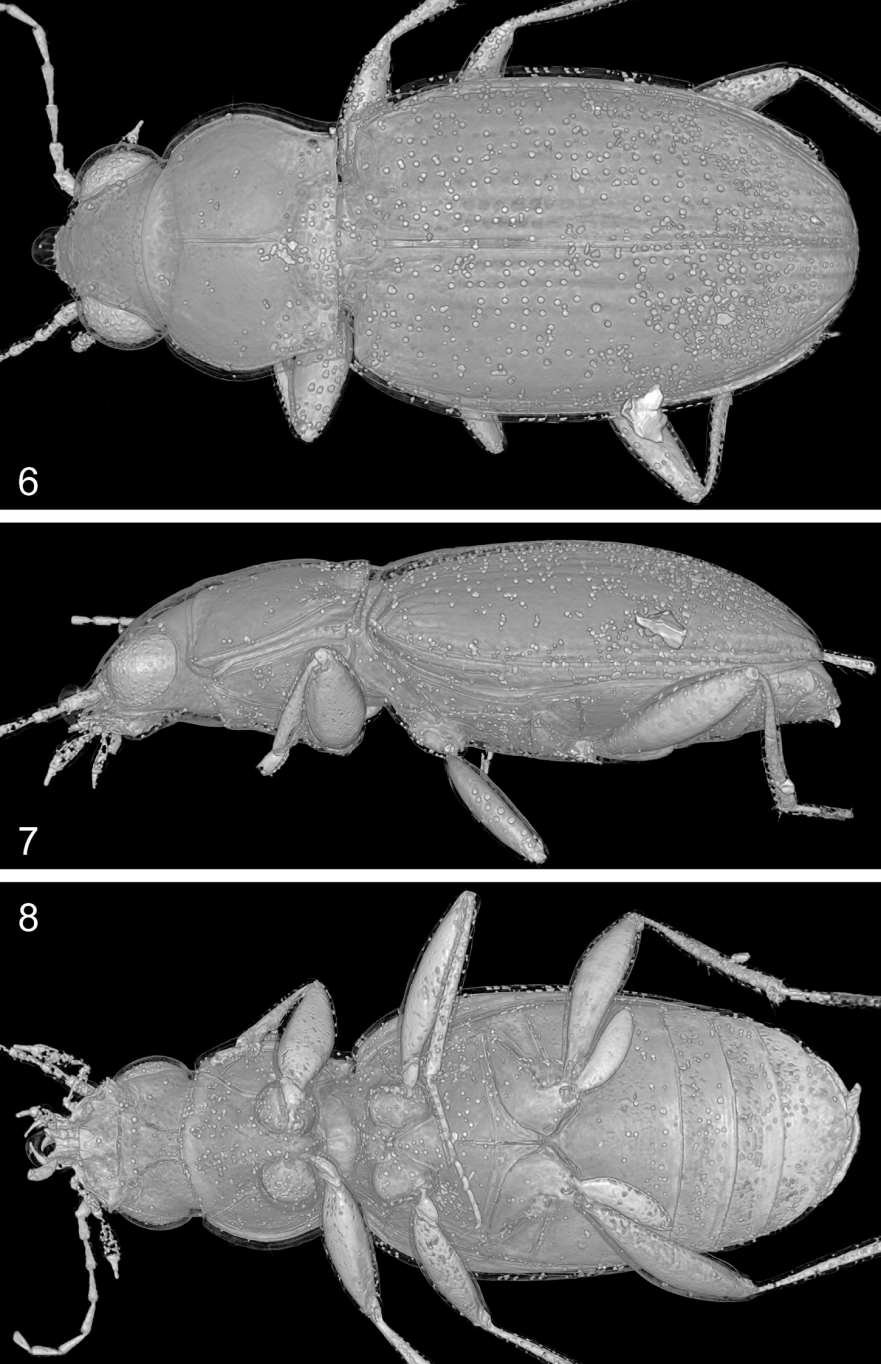
*Bembidion bukejsi* sp. n., volume rendering of the holotype. **6** dorsal aspect **7** left lateral aspect **8** ventral aspect.

The amber piece and its *Bembidion* fossil are in a comparatively very good conservation state. Most parts of the piece are clear, and the beetle body is well visible using light microscopy (Figs 1, 3–5). The mouth part is partly covered by milky coating and thus mandibles and maxillae cannot be investigated by light microscopy. The body is partly covered by minute particles (granules or tiny bubbles) of unknown origin. The exoskeleton of the specimen is partly slightly shrunken and thus dissociated from the inclusion wall (Figs 6–8). Four apical tarsomeres of the left proleg are lost. The aedeagus is well preserved and could be reconstructed together with its endophallic folding structures using micro-CT (Figs 12–17, 26).

#### Syninclusions.

One stellate hair and numerous dirt particles.

#### Description.

Body length: 3.5 mm.

Colour: The whole body surface appears blackish, very shiny, with metallic lustre; variation in colouration of the different parts of the beetle body is not recognizable.

Microsculpture: Surface of head including labrum with deeply engraved isodiametric sculpticells, pronotum with less deeply engraved slightly transverse meshes, elytral intervals with very finely engraved transverse meshes which are much smaller than on head and pronotum and which are not visible below magnification of ×100.

Head: Moderately large and transverse; length 0.75 mm, width 0.80 mm. Mandibles moderately slender. Labrum with apical margin slightly concave, dorsally with three pairs of setae near apical margin. Clypeus with one pair of setae in normal position. Shape and setation of maxillary palpi as typical for *Bembidion*, with apical segment subulate, approx. 2/5 of length of penultimate segment; penultimate segment markedly broadened towards apex. Antennae rather short, with pedicellus approx. 1.5 times longer than broad, and with two antennomeres extending beyond the pronotal base. Mentum and submentum distinct, mentum with medio-apical tooth simple, shortly rounded at tip, and with one pair of setae; pits absent. Eyes large, hemispherical, protruded; tempora very small, approx. 1/20 of eyes diameter, not visible in dorsal view. Disk moderately convex, smooth apart from the prominent microsculpture. Frontal furrows very shallow, very short, absent on disk. Supraorbital furrows flat, without punctures; two supraorbital setae present and in normal position for *Bembidion*.

Prothorax: Pronotum moderately large, length 0.76 mm, width 1.04 mm, transverse (width/length = 1.37), 1.3 times broader than head, subcordate, broadest slightly before middle, with sides faintly concave in posterior third. Laterobasal angles large, almost rectangular, not protruded laterally. Basal margin 1.1 times broader than apical margin. Disk moderately convex, smooth. Anterior margin finely convex in middle, lateroapical angles distinctly protruded, rounded. Posterior margin not beaded, slightly convex in middle, slightly sinusoidal towards laterobasal angles, latter very faintly shifted anteriad with respect to posterior margin of pronotum. Median longitudinal impression deep in middle, deepest before posterior transverse impression, but absent near pronotal apex and base; anterior transverse impression very shallow, smooth; posterior transverse impression moderately deep, smooth; laterobasal foveae large and rounded, moderately deep, smooth. Lateral gutter narrow throughout, smooth. Laterobasal carina long and straight, approx. 1/3 of length of pronotum. Both lateral and laterobasal setae present, with the lateral seta located slightly before middle of pronotum. Proepisternum glabrous, smooth.

Pterothorax: Elytra moderately convex on disc, in dorsal view narrow ovate, length 2.05 mm, width 1.37 mm, length/width = 1.50, widest near anterior third, distinctly wider than pronotum (width of elytra/width of pronotum = 1.32). Surface and lateral border glabrous and smooth apart from the primary elytral setation. Shoulders moderately broad with humeral margin angulate: The lateral bead forms an almost right angle with the abbreviated basal bead; the latter extends to the tip of the 4^th^ elytral stria. Crista clavicularis absent. Sides with preapical sinuation indistinct; subapical plica present. Parascutellar stria moderately long, parascutellar seta present. All striae complete, deeply impressed, impunctate, with intervals convex; apical stria (= common prolongation of the 5^th^, 6^th^, and 7^th^ striae) deeply impressed from level of the apical cross of 5^th^ and 6^th^ stria towards apex; recurrent stria lacking. Ninth interval moderately broadened from level of humeral umbilicate series towards apex. Each elytron with two discal setae in third interval, with relevant pores located close to, but separated from, third stria. Preapical seta located in the deepened apical portion of the seventh stria; the fine apical seta located at apical margin. Umbilicate series consist of eight setae: four humeral setae, with distance between first and second as well as second and third setae slightly larger than that between third and fourth setae; the fourth seta is located distinctly basad of the level of the anterior discal seta; both the subapical setae are markedly advanced and located at the beginning of the apical elytral third; two apical setae of the umbilicate series situated anterior of the junction of the eighth stria and lateral gutter. Metepisternum long, glabrous and smooth, with outer margin 1.6 times longer than anterior margin. Metasternal process without borders, moderately convex in middle. Hindwings fully developed.

Abdomen: Abdominal sternites V–VII each with one (male) pair of setae near apical margin; surfaces smooth, without hairs or micropunctures.

Legs: Relatively short, unmodified, femora moderately robust, protibiae straight and moderately dilated towards apex. First protarsomere markedly dilated, second protarsomere moderately dilated with apicolateral projection on inner margin.

Male genitalia (Figs 12–17, 26): Shape and size of median lobe as well as general structures of endophallus similar to species of the *Bembidion* subgenera *Bracteon* Bedel, 1879, and *Odontium* LeConte, 1848 (see Maddison 1993: 257): Median lobe moderately large, moderately slender, in lateral view moderately bent, with terminal lamella distinct, short tongue-shaped, slightly bent ventrad. Details of the parameres could not be recovered. Endophallus (in inverted positon) with a large ostidial flag with its internal tip distinctly bent back to the dorsal side of median lobe; dorsal field of ostium distinct; dorsal plate very large with left side more than right side. Below the dorsal plate an extended folding structure is developed (central fold system) which covers the central sclerite and the brush sclerite complexes dorsally and, which proceed in the flagellum distally; latter rather short, almost straight, not extending to the dorsal tip of the ostidial flag. Central and brush sclerite complexes both moderately large and situated side by side in middle portion of median lobe, overlaying each other in lateral view; central sclerite with left lobe lenticular and with right lobe large, situated more basad; brush sclerite apico-ventrally markedly prolonged with complicate folding structures.

**Figures 9–13.**
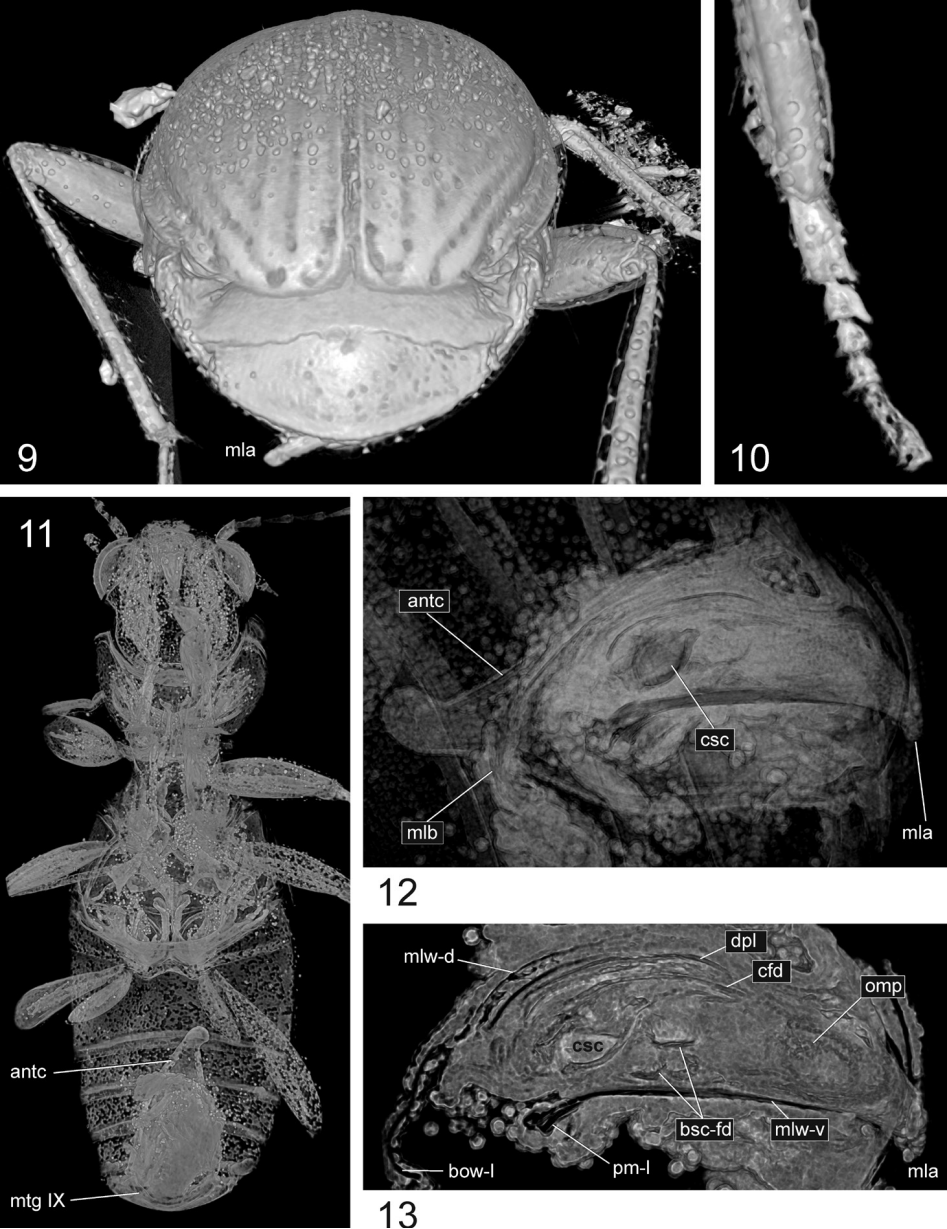
*Bembidion bukejsi* sp. n., holotype, volume rendering of selected body parts **9** caudal aspect of body **10** tarsomeres and distal portion of tibia of right proleg. **11** frontal section of body (ventral aspect) showing position of the abdominal segment IX which surrounds the aedeagus **12** abdominal segment IX and aedeagal median lobe, left lateral aspect **13** sagittal section of aedeagal median lobe, left lateral aspect. Abbreviations: antc = antecosta; bow-l = left wall of basal orfice; bsc-fd = folding structures originating from the endophallic brush sclerite; cfd = central folding system of endophallus; csc = central sclerite of endophallus (left lobe); dpl = dorsal plate of endophallus; mla = aedeagal median lobe apex; mlb = aedeagal median lobe base; mlw-d = dorsal wall of median lobe; mlw-v = ventral wall of median lobe; mtg IX = mediotergite IX; omp = ostial microtrichial patch; pm-l = left paramere (basal portion).

**Figures 14–17.**
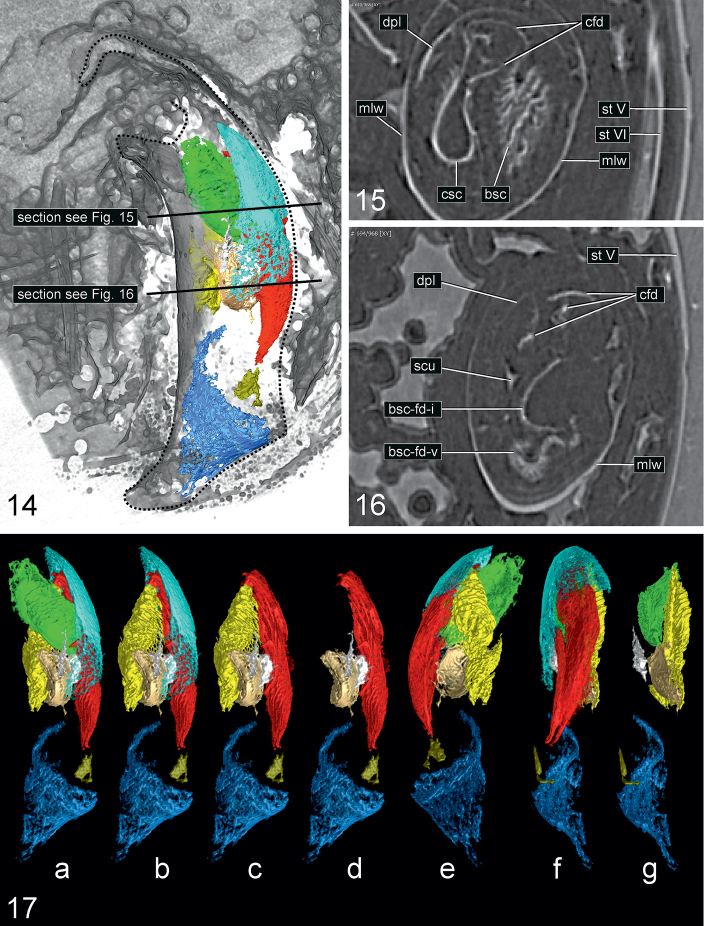
*Bembidion bukejsi* sp. n., holotype, visualization of endophallic structures using micro-CT. **14** volume rendering of the aedeagal median lobe with sclerotized endophallic structures highlighted in colors **15–16** parts of transverse sections through the abdomen with aedeagus (for position of slices see Fig. 14) **17** sclerotized endophallic structures separated and highlighted in colors using serial sectioning (a-d, left lateral aspect; e, right lateral aspect; f-g, dorsal aspect). Colour coding: beige = internal fold originating from the brush sclerite; green = central sclerite; khaki = dorsal field; light blue = dorsal plate; marine blue = ostidial flag; red = central fold system; white = unknown sclerite; yellow = brush sclerite. Abbreviations: bsc = brush sclerite; bsc-fd-i = internal fold originating from the brush sclerite; bsc-fd-v = ventral prolongation of the brush sclerite; cfd = central folding system; csc = central sclerite; dpl = dorsal plate; mlw = wall of median lobe; scu = unknown sclerite; st = sternite.

#### Derivatio nominis.

The species epithet is a dedication to the collector Andris Bukejs, which kindly allowed us to investigate this unique specimen.

#### Relationships and recognition.

The angulate humeral margin together with the position of the elytral discal setae separated from third stria and the shape of the endophallic structures provide important evidence for probable relationships of the fossil species. An angulate humeral margin is also developed in species of the *Bembidion* sensu lato lineages *Andrewesa* Netolitzky, 1931, *Hoquedela* Müller-Motzfeld, 1988, *Peryphophila* Netolitzky, 1939, *Phyla* Motschulsky, 1844, *Plataphodes* Ganglbauer, 1891, as well as in the *Hydrium* and *Odontium* complexes sensu Maddison (2012). *Pekinium* Csiki, 1901, is another taxon described based on a species with angulate humeral margin, however, the type specimen seems lost and the state of the taxon remain questionable (Toledano and Sciaky 1998) and will thus not further considered here.

**Figures 18–26.**
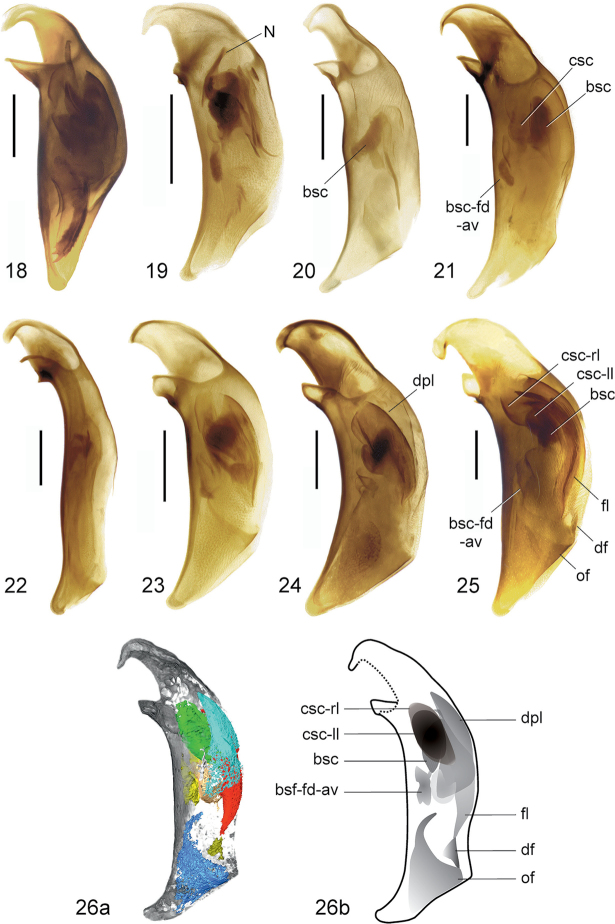
Aedeagal median lobes and endophallic structures of recent Bembidiina (**18–25**) and the fossil *Bembidion bukejsi* sp. n. (**26**), left lateral view. **18** *Hoquedela k. kirschenhoferi* Müller-Motzfeld, 1988 **19** Bembidion (Phyla) tethys Netolitzky, 1926 **20** B. (Plataphus) f. fellmanni Mannerheim, 1823 **21** B. (Melomalus) altaicum Gebler, 1833 **22** B. (Peryphophila) eurydice Andrewes, 1926 **23** B. (Andrewesa) patris Schmidt, 2010 **24** B. (Bracteon) lapponicum Zetterstedt, 1828 **25** B. (Odontium) striatum Fabricius, 1792 **26** B. (Eodontium) bukejsi subgen. n., sp. n. (Fig. 26a, volume rendering with highlighted structures, see also Fig. 14; Fig. 26b, schematic reconstruction of putative organization of endophallic structures). Scale bar: 2.2 mm. Abbreviations: bsc = brush sclerite; bsc-fd-av = apico-ventral prolongation of the brush sclerite; csc = central sclerite; csc-ll = left lobe of central sclerite; csc-rl = right lobe of central sclerite; df = dorsal field; dpl = dorsal plate; fl = flagellum of the central fold system; N = N-sclerite (Maddison 2012); of = ostidial flag.

Based on a comprehensive phylogenetic analysis of *Bembidion* and related ground beetles, Maddison (2012) has shown that the character state ‘angulate humeral margin’ evolved probably five times independently within *Bembidion* sensu lato. In *Phyla*, the abbreviated basal bead extends to the tip of the 5^th^ elytral stria and is therefore distinctly shorter than in *B. bukejsi* sp. n.. The same holds true for *Peryphophila*, which was not included in the analysis of Maddison (2012). Species of both taxa can be additionally distinguished from *B. bukejsi* sp. n. in the elytral discal setae, which are situated in the third stria, and by its very different endophallic morphology. In *Phyla*, an additional endophallic sclerite is developed basad of the central sclerite complex (probably homolog to the “N-sclerite” of the *Notaphus* series sensu Maddison 2012, Fig. 19), the central sclerite is irregularly shaped, and the dorsal field is lacking. In *Peryphophila*, the central sclerite is markedly small and the aedeagal median lobe is much more slender compared to *B. bukejsi* sp. n. (Fig. 22). In the *Plataphus* complex sensu Maddison (2012), including *Plataphodes*, the central sclerite is only hardly sclerotized or sometimes lacking and thus, the endophallic structures are very differently developed (Fig. 20) compared to the situation in *B. bukejsi* sp. n.. Moreover, also in species of the *Plataphus* complex, the elytral discal setae are situated in the third stria. In the High Asian taxon *Hoquedela*, the elytral discal setae are separated from the third stria similarly to the organization in *B. bukejsi* sp. n.. However, *Hoquedela* differs from *B. bukejsi* sp. n. by the shape of the aedeagal median lobe (Fig. 18), which is markedly robust with a swollen middle portion and very stout apex, by an extensively sclerotized folding structure of endophallus particularly near median lobe ostium, and by modified mesotibia which are suggestively sinuate due to slightly convex interior surface in middle of tibia and slightly inwardly bent tibial apex.

The external shape of the aedeagal median lobe and sclerotization patterns of the endophallic structures present in *B. bukejsi* sp. n. resemble those of species of *Andrewesa* (Fig. 23), the *Hydrium* complex and *Odontium* series (Figs 24, 25, Maddison 1993: Figs 112–128), as well as in *Liocosmius* Casey, 1918 (Maddison and Cooper 2014: Fig. 12), *Melomalus* Casey, 1918 (Fig. 21), *Trechonepha* Casey, 1918, *Trichoplataphus* Netolitzky, 1914 (Toledano and Schmidt 2010: Figs 33–42), and several species of the *Ocydromus* series sensu Maddison (2012). The common male genital characters of most species of these lineages are: (i) median lobe rather slender, slightly bent throughout, with (ii) terminal lamella short tongue-like, not bent backwards; (iii) endophallic central sclerite and brush sclerite complexes both moderately large and (in inverted position) situated side by side in middle portion of median lobe, overlaying each other in lateral view; (iv) central sclerite with left lobe lenticular and (v) with right lobe large and situated more basad; (vi) brush sclerite apico-ventrally markedly prolonged; (vii) dorsal plate largely developed and (viii) without additional sclerites near basal opening of median lobe. The multi-gene analyses presented by Maddison (2012) support a monophyly of all these groups together with the *Plataphus* complex (in the following called the “*Odontium*-*Plataphus*-*Ocydromus* (OPO) clade). Within this highly diverse clade, a natural group ‘*Ocydromus* series plus *Plataphus* complex plus *Trichoplataphus*’ is recovered, however, the relationships of the other main lineages remain unresolved by the molecular data. In three species groups of the OPO clade, the character states ‘angulate humeral margin’ and ‘elytral discal setae are separated from the third stria’ resemble the organization observed in the fossil *B. bukejsi* sp. n., *Andrewesa*, the *Hydrium* and the *Odontium* complexes.

Molecular data suggest that *Andrewesa* is the sister group of the *Hydrium* complex, while the *Odontium* complex is the sister group of the *Hydriomicrus* complex; both the latter taxa together form the *Odontium* series (Maddison 2012). The *Hydriomicrus* complex includes species with rounded humeral margin and elytral discal setae situated in the third stria. Close relationships of the *Hydrium* complex plus *Andrewesa* with the *Odontium* series, are not supported by the molecular data. Thus, it remains unclear whether the character states ‘angulate humeral margin’ and ‘elytral discal setae separated from third stria’ were evolved in the OPO clade only once or even twice; in latter case, an independent evolution of these features in the *Hydrium* complex plus *Andrewesa*, and in the *Odontium* complex of the *Odontium* series has to be assumed.

We could not find additional derived characters justifying a further assignment of the fossil species *B. bukejsi* sp. n. with one of these three lineages of the OPO clade. Similarities with representatives of extant species groups are considered to be symplesiomorphies, e.g., the shape of the pronotum and the development of the elytral striation. In *B. bukejsi* sp. n., the pronotal basolateral foveae are large and rounded as in *Andrewesa*. In many species of the *Hydrium* complex and in all species of the *Odontium* series, the basolateral foveae are linear impressed, and the area between foveae and laterobasal carina is markedly wide and convex. The latter represent apomorphic states. The elytral striae are deeply impressed throughout in *B. bukejsi* sp. n., and likewise in *Hydriomicrus* Casey, 1918, *Hirmoplataphus* Lindroth, 1963, and *Pseudoperyphus* of the *Odontium* series. In all the remaining lineages of the *Odontium* series as well as in *Andrewesa* and the *Hydrium* complex, the external elytral striae are more or less distinctly more shallowly impressed before the apex. Latter is considered an apomorphic character state.

Due to the impunctate elytral stria *B. bukejsi* sp. n. differs strikingly from all species of *Andrewesa*, the *Hydrium* complex and the *Odontium* series. However, impunctate or indistinctly punctate elytral striae are also present in other lineages of the clade, e.g., *Melomalus*, the *Ocydromus* series, and the *Plataphus* complex. This character shows also continuous variation in other Bembidiina clades and is therefore not informative with regard to its phylogenetic implications.

Due to the peculiar combination of the character states observed in *B. bukejsi* sp. n., we conclude that this fossil species represents an extinct lineage of the OPO clade, probably related to either *Andrewesa* plus the *Hydrium* complex or the *Odontium* series, or to both of these sub-clades. Given the present state of knowledge, the assignment to one of the known subgeneric taxa is impossible and therefore we decided to describe a new subgenus for this fossil representative of *Bembidion* (see below).

#### Remarks on biogeography and ecology.

The above mentioned OPO clade represents one of the most species rich clades of *Bembidion* sensu lato (Maddison 2012) with hundreds of recent species occurring mainly in the Holarctic region and, in much smaller number, also in the Oriental region and Africa. Species of the OPO clade are adapted to very different climates (tropical: e.g., *Microserrullula*, to arctic: e.g., some species of *Plataphus*), and prefer very different habitats although most of them are ripicolous or paludicolous. The occurrence of *B. bukejsi* sp. n. in the Baltic amber forest as the first fossil representative of the OPO clade is therefore in accord with the expected distribution of that clade during the Early Cenozoic.

### Eodontium subgen. n.

Taxon classificationAnimaliaColeopteraCarabidae

http://zoobank.org/9A9BCB24-EDCA-44DB-B1FB-57AEC4033F56

#### Type species.

*Bembidion bukejsi* sp. n.

#### Derivatio nominis.

The new subgenus name is an abbreviated combination of “Eocene” and “Odontium” and thus combines the period of the Earth history when the type species of the new subgenus lived, with the name of a probably related subgeneric taxon of *Bembidion*.

#### Differential diagnosis.

An extinct lineage of the OPO clade based on the combination of the seven male genital characters mentioned in the description of *B. bukejsi* sp. n. above. From most lineages of this highly diverse clade, Eodontium subgen. n. is distinguished by the presence of an angulate humeral margin and by the elytral discal setae, which are separated from the third stria. From all species of the subgenus Andrewesa and of the *Hydrium* and *Odontium* complexes which have these character states similarly or identically developed, Eodontium subgen. n. is easily distinguished by the impunctate elytral stria. The Eocene lineage differs additionally from *Andrewesa* by the thoroughly deeply impressed elytral striae and by the lack of elytral transverse depressions, and from the *Hydrium* and *Odontium* complexes by the large, roundish, deeply impressed laterobasal foveae of the pronotum, which is also distinctly less bulged on disc. Moreover, most species of the latter complexes differ markedly by the shape of pronotum, which has a base distinctly broader than apical margin and basolateral area between foveae and side margin convex.

An angulate humeral margin is also developed in High Asian subgenus Peryphophila and some species of the Holarctic *Plataphus* complex. However, in these lineages of the OPO clade the elytral discal setae are situated in the third stria. In addition, the endophallic central sclerite complex is much smaller compared to Eodontium subgen. n.

### Bembidion alekseevi sp. n.

Taxon classificationAnimaliaColeopteraCarabidae

http://zoobank.org/A58EB208-0710-496E-85ED-3D6D9E4AF9F9

Figs 28 , 29–31 , 32–34 , 35–36

#### Holotype.

Female in Baltic amber; size of amber piece approximately 14 × 12 × 4 mm (irregularly cut, Fig. 31), with collection label data “AWI 130”, in Vitalii I. Alekseev Collection, Kaliningrad. This amber piece was found in the surf zone of the Baltic Sea coast of the Sambian peninsula, in winter 2014-2015.

#### Preservation status.

The amber piece is permeated by broad light-reflecting flow lines particularly at level of the *Bembidion* inclusion. The anterior portion of the ventral side of the beetle body is covered by milky coating. Thus, mouth parts, ventral side of prothorax, and some portions of the dorsal surface of the fossil cannot by investigated by light microscopy (Figs 29–30). The specimen is markedly shrunken with its exoskeleton which is disintegrated, shattered, and dissociated from the inclusion wall (Fig. 35). However, the negative imprint of the beetle body (= inclusion wall) could be recovered in detail using micro-CT (Figs 32–36). The left elytron is somewhat impressed behind the middle which is probably the result of the embedding of an immature and thus teneral specimen (Fig. 32). The hindwings are inflated caudal of the elytra (Fig. 30).

**Syninclusions.** Few dirt particles, numerous air bubbles.

**Description.** Body length: 3.9 mm.

Colour: Head and pronotum blackish brown with marked metallic lustre. Elytra middle brown with yellowish side margin (eighth and part of the seventh intervals), a small yellowish pre-apical spot between sixth and eighth interval at the beginning of the apical elytral third, and additionally with small yellowish spots in the third (4 spots) and fifth intervals (3 spots) as shown in Fig. 28.

**Figures 27–28.**
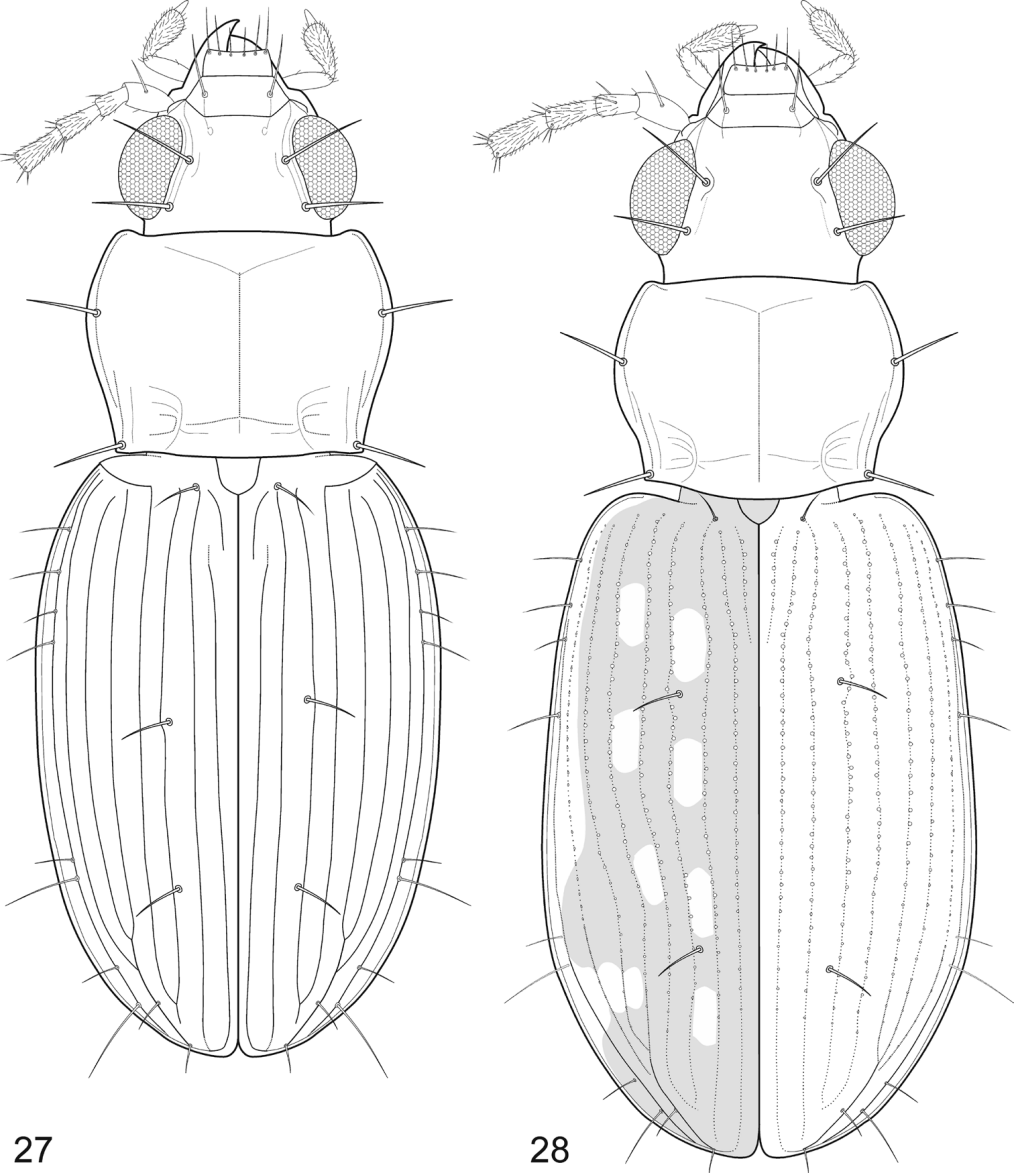
Fossil *Bembidion* Latreille, 1802, reconstruction of the external shape in dorsal view. **27** *B. bukejsi* sp. n. **28** *B. alekseevi* sp. n.

Microsculpture: Surfaces of head including labrum and pronotum with deeply engraved isodiametric sculpticells. Elytral intervals with very finely engraved, small and irregularly formed meshes which are not clearly visible below magnification of ×100.

Head: Moderately large and transverse; length 0.84 mm, width 0.91 mm. Mandibles moderately stout. Labrum with apical margin slightly concave, dorsally with three pairs of setae near apical margin. Clypeus with one pair of setae in normal position. Apical segment of maxillary palpus subulate, approx. 2/5 of length of penultimate segment; penultimate segment rather long and slender, moderately dilated anteriorly. Antennae moderately slender, with pedicellus almost two times longer than broad, and with four antennomeres extending beyond the pronotal base. Mentum and submentum distinct (shape and setation of medio-apical tooth could not be recovered); pits absent. Eyes large, hemispherical protruded; tempora very small, approx. 1/16 of eyes diameter. Disk slightly convex, smooth apart from the prominent microsculpture. Frontal furrows very shallow, very short, absent on disk. Supraorbital furrows very shallow, without punctures; two supraorbital setae present and in usual position for *Bembidion*. The pore of the anterior supraorbital seta is semicirculary surrounded by a prominent ridge on its internal side (Fig. 36).

**Figures 29–31.**
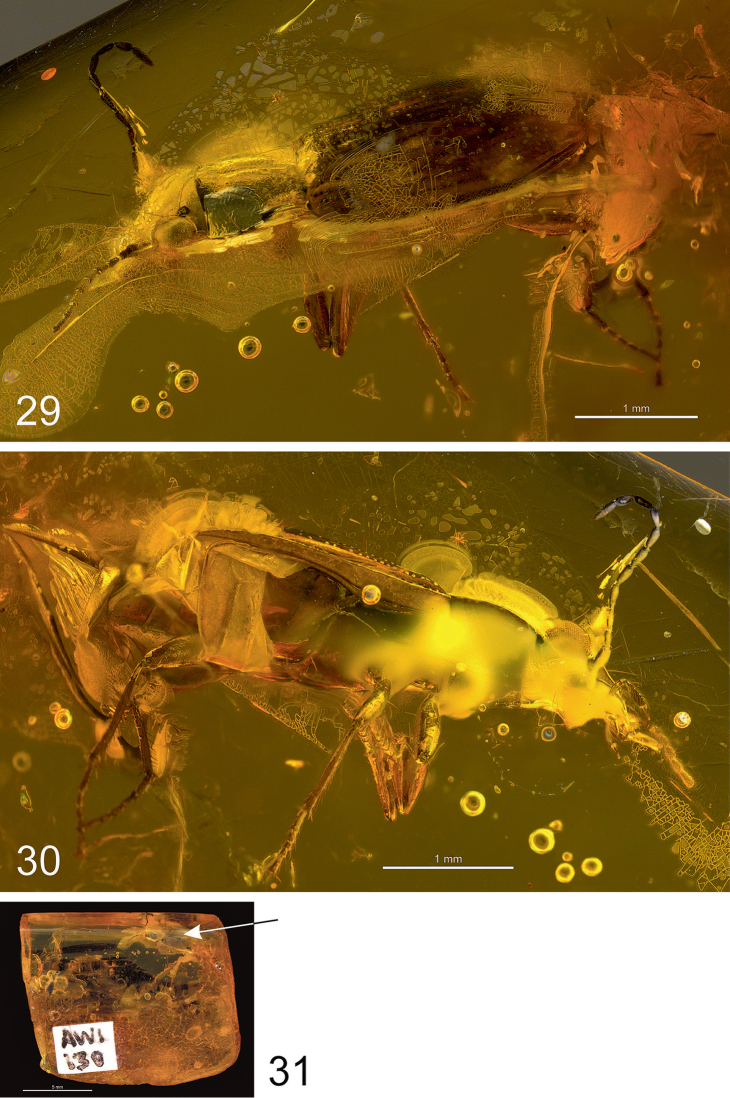
*Bembidion alekseevi* sp. n., light microscopic images of the holotype. **29** left dorso-lateral aspect **30** right ventro-lateral aspect **31** general view of the amber piece; the position of the fossil is marked by an arrow.

Prothorax: Pronotum rather small, length 0.77 mm, width 1.02 mm, transverse (width/length = 1.32), 1.1 times broader than head, subcordate, broadest in middle, with sides distinctly concave in posterior third. Laterobasal angles large, slightly obtuse, not protruded laterally. Basal margin 1.05 times broader than apical margin. Disk moderately convex, smooth apart from the prominent microsculpture. Anterior margin slightly convex in middle, lateroapical angles small and rounded, slightly protruded. Posterior margin not beaded, distinctly convex in middle and concave near laterobasal angles. Median longitudinal impression moderately deep in middle, absent near pronotal apex and base; anterior and posterior transverse impressions very shallow, smooth; laterobasal foveae large and rounded, moderately deep, smooth. Lateral gutter narrow and flat, faintly widened in middle, smooth. Laterobasal carina long and straight, approx. 1/3 of length of pronotum. Both lateral and laterobasal setae present, with the lateral seta located slightly before middle of pronotum. Surface structures on ventral side of the prothorax could not be imaged.

**Figures 32–34.**
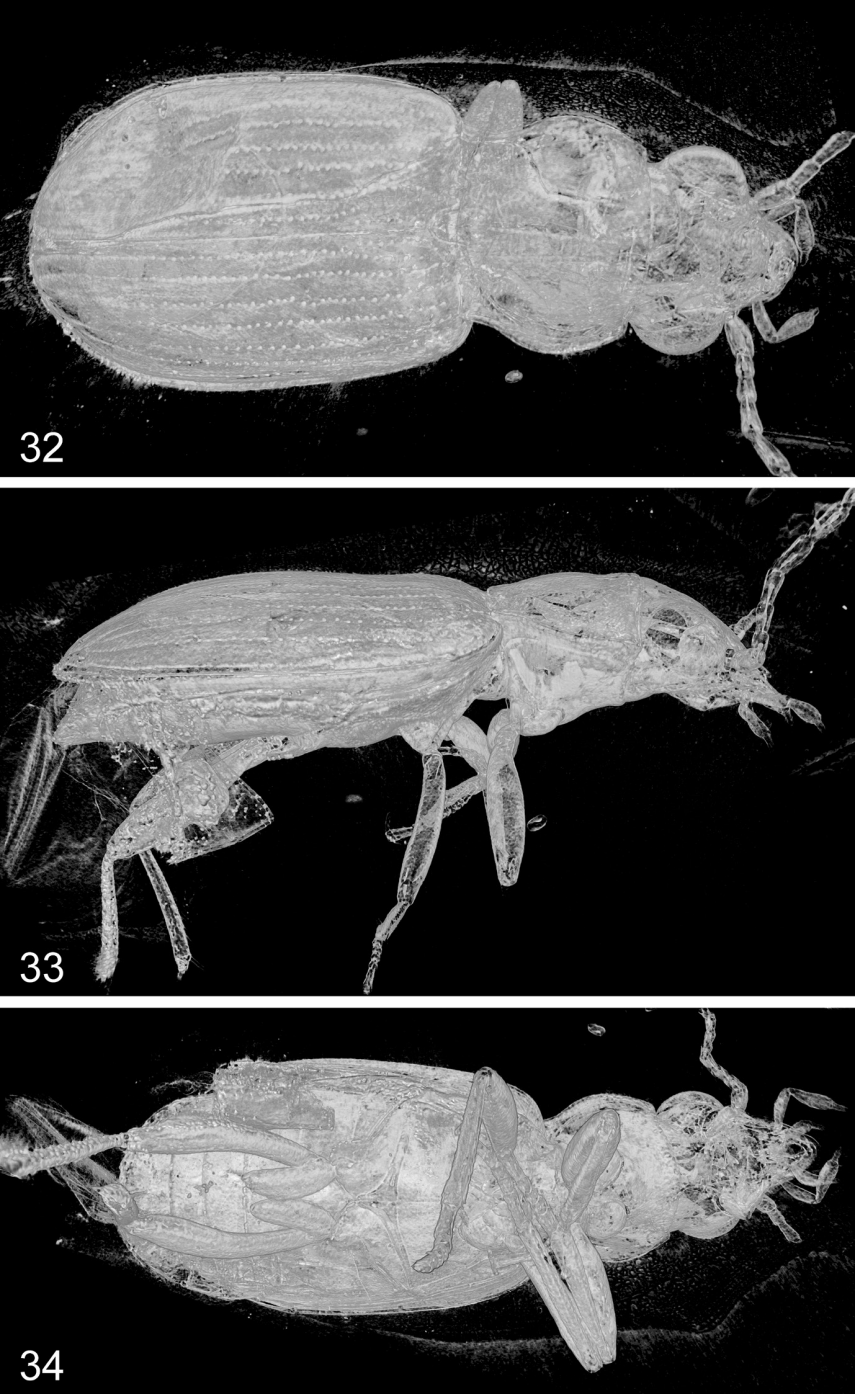
*Bembidion alekseevi* sp. n., volume rendering of the holotype. **32** dorsal aspect **33** right lateral aspect **34** ventral aspect.

Pterothorax: Elytra in lateral view moderately convex, slightly flattened on disc, in dorsal very slender ovate, length 2.34 mm, width 1.52 mm, length/width = 1.54, widest slightly behind the middle, distinctly wider than pronotum (width of elytra/width of pronotum = 1.49). Surface and lateral border glabrous and smooth apart from the primary elytral setation. Shoulders moderately broad, humeral margin rounded, sides with preapical sinuation indistinct. Presence or absence of crista clavicularis as well as subapical plica could not be imaged. Parascutellar stria long, parascutellar seta present. All striae complete, slightly impressed but markedly punctate, intervals flat or slightly convex; apical stria deeply impressed from the apical cross of 5^th^ and 6^th^ stria towards apex; recurrent stria lacking. Ninth interval very narrow in front, slightly broadened from beginning of apical third towards apex (the left elytron of the specimen is artificially flattened in anterior third and therefore, the external intervals appearing distinctly broader caudally than on the right elytron, see Figs 19, 31). Each elytron with two discal setae in third interval, with relevant pores distinctly separated from third stria. Preapical seta located in the deepened apical portion of the seventh stria; the fine apical seta located at apical margin. Subapical setae of umbilicate series could not be imaged; the humeral series consist of four setae, with distance between first and second setae slightly larger than between second and third setae, and with distance between third and fourth setae distinctly larger than between first and second setae; the fourth seta located slightly caudad of the level of the anterior discal seta; two apical setae of the umbilicate series situated anterior of the junction of the eighth stria and lateral gutter. Metepisternum markedly long, glabrous and smooth, with outer margin 2.3 times longer than anterior margin. The surface of the metasternal process could not be imaged. Hindwings fully developed.

**Figures 35–36.**
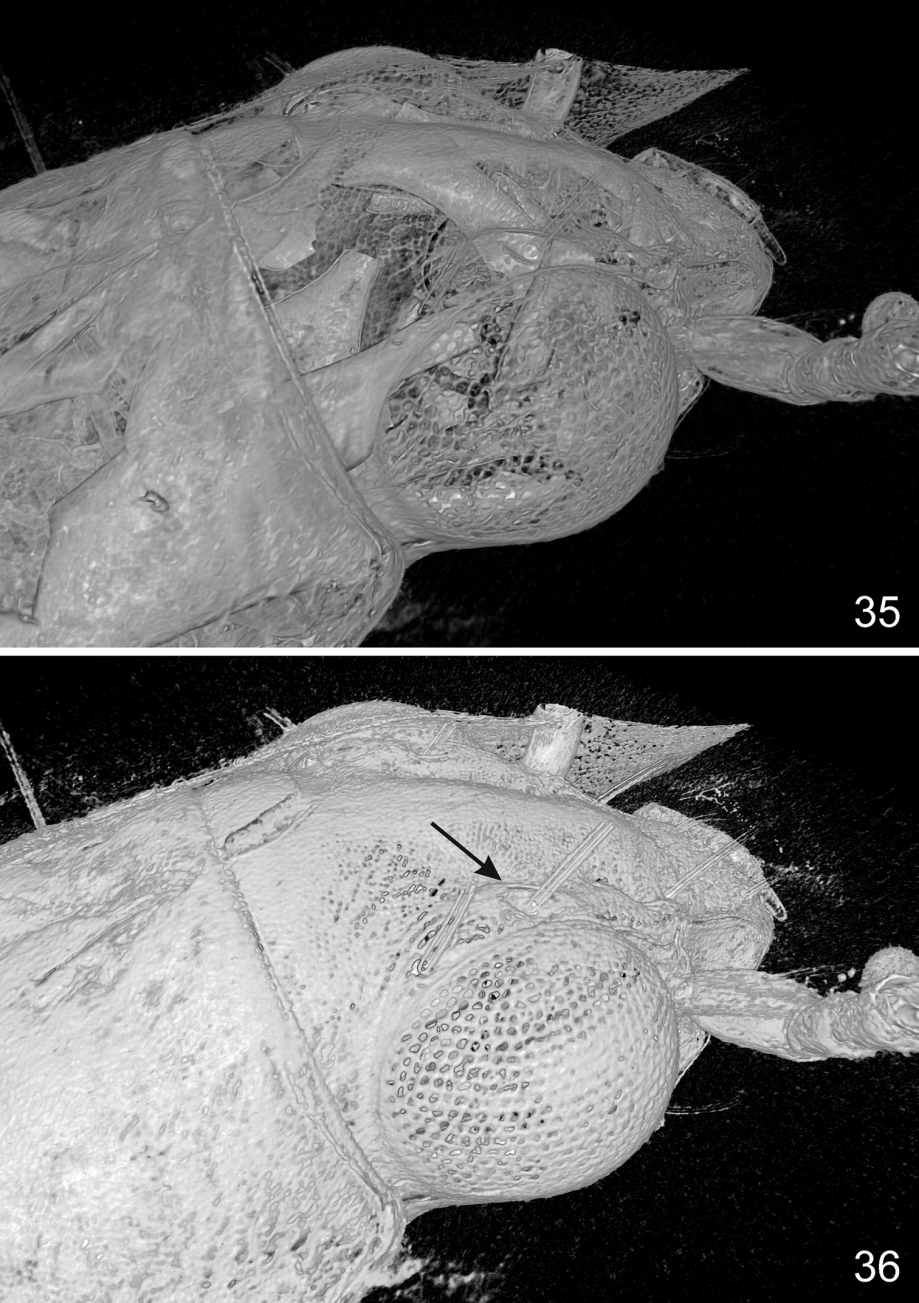
*Bembidion alekseevi* sp. n., volume rendering of the head capsule of the holotype using different grayscale thresholds, dorsal aspect. The arrow in Fig. 36 point to the prominent ridge on internal side of the anterior supraorbital pore, which is characteristic for representatives of the Bembidion subgenus Eupetedromus.

Abdomen: Abdominal sternites V–VI with one, VII with two (female) pairs of setae near apical margin; surfaces smooth, without hairs or micropunctures.

Legs: Moderately short, unmodified, femora moderately robust, protibiae straight and moderately dilated towards apex.

Female genitalia: Could not be recovered using micro-CT.

#### Derivatio nominis.

The new species is dedicated to Vitalii I. Alekseev, Kaliningrad, for his important contributions to the systematics and biogeography of Cenozoic Coleoptera.

#### Relationships and recognition.

This fossil *Bembidion* is considered to be a representative of the subgenus Eupetedromus Netolitzky, 1911 based on combination of the following characters: (i) micro-meshes on surface of head and pronotum deeply engraved and thus contrasting with the elytra which appearing polished when viewed by magnification below x80; (ii) penultimate segment of maxillary palpus rather slender; (iii) anterior supraorbital pore on internal side semicirculary surrounded by a prominent ridge; (iv) supraorbital furrows very shallow; (v) pronotal median longitudinal impression not deepened near base; (vi) pronotal posterior transverse impressions very shallow; (vii) elytral discal setiferous pores distinctly separated from third stria; (viii) distance between third and fourth setae of the umbilical humeral series much larger than between first and second and between second and third setae. Similar patterns of elytral chaetotaxy are also observed in other species groups of *Bembidion*, e.g., *Emphanes* Motschulsky, 1850, *Notaphemphanes* Netolitzky, 1920, *Notaphocampa* Netolitzky, 1914, *Notaphus* Dejean, 1821, *Omotaphus* Netolitzky, 1914, *Talanes* Motschulsky, 1864, *Trepanedoris* Netolitzky, 1918, *Trepanes* Motschulsky, 1864, and the *Diplocampa* complex sensu Maddison (2012). Species of the latter complex differ from this fossil and other species of *Eupetedromus* by presence of long and deep frontal and suborbital furrows. The same feature applies for species of the subgenera *Emphanes*, *Notaphemphanes*, *Talanes*, *Trepanedoris*, and *Trepanes* which additionally differ by much more convex discs of head and pronotum, weakly engraved microsculpture on head and pronotum and, in most cases, by stouter penultimate maxillary palpomeres and deeper median longitudinal and posterior transverse impressions of pronotum. Species of *Notaphus* are very similar to the fossil *B. alekseevi* sp. n. last but not least due to the characteristic colour patterns of the body. However, species of *Notaphus* lack the prominent ridge on internal side of the anterior supraorbital pore which is observed in *B. alekseevi* sp. n. Most species of *Notaphocampa* and *Omotaphus* differ strikingly by very different patterns of its elytral microsculpture; in the Ethiopian species *B. scotti* Netolitzky, 1931 the micro-meshes on the elytra are extremely fine engraved similar to what is observed in *B. alekseevi* sp. n. (see Bonavita et al. 2016), however, in *B. scotti* the microsculpture on pronotal surface is likewise weak and thus very different from what is developed in *B. alekseevi* sp. n.

#### Remarks on biogeography and ecology.

The subgenus Eupetedromus contains ten species which are distributed in the temperate and boreal zones of the Holarctic region (Marggi et al. 2003, Lorenz 2005, Bousquet 2012). The occurrence of *B. alekseevi* sp. n. in the Baltic amber forest is therefore in accordance with the expected distribution of *Eupetedromus* group during the Early Cenozoic if one would accept presence of extra-tropical habitat conditions at least locally in the area of this forest. Based on comparative studies, Wolfe (1975) found close relationships of the flora of the Eocene northern Europe to the modern flora of Indo-Malaya. This flora represents a mixture of tropical/subtropical and temperate elements with occurrences of the latter along slopes of the many high elevated areas of this region. Based on evidences from insect fossils, Ander (1942), Schmidt and Faille (2015), and Schmidt et al. (2016b) suggested the presence of higher elevated areas in the Baltic amber forests which may represent suitable habitats for insects adapted to the temperate climate, e.g., *Eupetedromus* ground beetles.

## Conclusions

At least three species of *Bembidion* are currently known to occur in the Baltic amber forests, which can be assigned to very different lineages of the genus (phylogeny based on Maddison, 2012). Thus, the mega-diverse genus *Bembidion* was likely already rich in species during the Eocene. Furthermore, this suggests that the discovery of additional *Bembidion* species fossilized in Paleogene deposits is very likely.

The assignment of the fossil species to certain *Bembidion* lineages results in preliminary conclusions regarding the habitat preferences of the fossil taxa and the ecological conditions in the Amber forests. Since extant species of the subgenus Philochthemphanes possess a semi-arboreal life style and are adapted to very humid forests with high density of epiphytes in the shrub and tree layers, it seems very likely that similar conditions were present in the Baltic Amber forests and that *B. christelae* was a likewise semi-arboreal beetle, which could have been easily trapped by the freshly leaked resin while climbing on the tree bark.

All extant *Philochthemphanes* species are adapted to temperate climates. Although the distribution of this subgenus in East Asia reaches far into tropical latitudes, occurrences of *Philochthemphanes* in these regions are restricted to high mountains and consequently, to the corresponding colder temperature conditions of the respective local climate. In addition, species of the proposed sister group, the subgenus Philochthus, as well as B. (Lindrochthus) wickhami, which is closely related to the *Philochthus*-*Philochthemphanes* clade based on molecular data (Maddison 2012), are adapted to temperate climates and not present in the tropical zone. Temperate climatic conditions may have thus existed at least locally in the region of Eocene Baltic amber forests. Similar conclusions can be drawn from the occurrence of the holarctic subgenus Eupetedromus in the Baltic Amber forest, which are now present in the warm to cold temperate zones. If we assume that the proposed placement of the fossil *B. alekseevi* sp. n. into this species group is correct, the occurrence of *Eupetedromus* taxa in the Baltic Amber forest would be another strong indication of temperate climatic conditions in the Eocene Northern Europe.

The general assumption that the Baltic Amber forest occurred in the paratropical to subtropical zone of the Eocene (Weitschat and Wichard 2010) is in striking contrast to the frequent occurrence of temperate species in the Baltic amber fossil record. One explanation for the presence of such species is the existence of hypothetical highly elevated mountain ranges in the Eocene of North Europe (Ander 1942, Kohlman-Adamska 2001, Schmidt et al. 2016a). This hypothesis is supported by the discovery of several tiny wingless and even blind ground beetles in Baltic amber, which might have been adapted to the high altitudes (Schmidt and Faille 2015, Schmidt et al. 2016a, b). However, Alekseev and Alekseev (2016) concluded from a data analysis of vascular plants and beetles preserved in Baltic amber that this forest was formed on a plain or slightly hilly area. Another hypothesis was presented by Archibald & Farrell (2003): Low temperature seasonality with milder winters may have caused the presence of clearly thermophilic organisms in the higher latitudes during the Eocene, but not necessarily tropical or subtropical climate. Consequently, the ecology of the Baltic amber forest is still not being fully understood and needs further investigations based on a significantly improved fossil record. Modern investigation techniques, particularly X-ray microscopy, enable a much more sophisticated analysis of fossils including internal structures, consequently revealing more informative characters for a robust taxonomic placement of the fossils especially with regard to affinities to extant lineages. Last but not least, based on knowledge of the ecology and distribution of the recent representatives, a better understanding of the systematic position of the fossils provide a unique possibility to infer to the corresponding habitat of the respective Eocene species.

## Supplementary Material

XML Treatment for Bembidion succini

XML Treatment for Bembidion christelae

XML Treatment for Bembidion bukejsi

XML Treatment for Eodontium

XML Treatment for Bembidion alekseevi
